# The imperative of professionalising healthcare management: A global perspective

**DOI:** 10.1016/j.fhj.2024.100170

**Published:** 2024-08-08

**Authors:** Qasem Al Salmi, Jehan Al Fannah, Eric de Roodenbeke

**Affiliations:** a Directorate General of Planning, Ministry of Health, Muscat, Oman; b Quality and Patient Safety Department, Royal Hospital, Muscat, Oman; c International Health Services Management, Nantes, Pays de la Loire, France

**Keywords:** Professionalisation, Management, Leadership, Sustainable development, Global healthcare

## Abstract

Effective healthcare management for addressing complex organisational challenges is crucial for efficient healthcare delivery. Healthcare management involves organising, coordinating, planning and operationalising healthcare services, as well as leading people to ensure the delivery of effective patient care. Healthcare management applies management principles and practices to various healthcare organisations, such as hospitals, functional departments, clinics, cross-functional departments and public health organisations. Recognising a gap in management training, especially for clinicians having managerial responsibilities, is a call for global professionalisation of healthcare management to equip leaders with essential skills. In many healthcare settings across the globe, healthcare management does not always require professional management qualifications. This article advocates for the need for a structured approach towards professionalising healthcare management globally and especially in the Eastern Mediterranean Region (EMR).

## Introduction

Peter Drucker described hospitals as the most complex organisational challenge humanity has ever undertaken.^1^ In many healthcare systems, the responsibility for running the departments, overseeing hospitals, policymaking and regulatory compliance primarily falls upon physicians and other healthcare professionals.^2^ Unfortunately, these professionals often lack the appropriate training and structured development necessary to effectively deliver high-quality and sustainable healthcare services.^3^ To bridge this critical gap in healthcare management expertise, many countries have introduced in-service training and mentoring programmes. While these programs offer immediate relief, there remains a pressing need for a comprehensive and strategic approach to nurturing proficient healthcare managers.^3^

### What is healthcare management professionalisation about?

In the healthcare industry, most of the staff are recognised as belonging to a profession^4^ requiring extensive education and qualifications. A profession has a well-defined scope of activities, most often backed up by regulations and laws. However, those practising healthcare management have not been universally granted the same status. In medicine, obtaining a professional qualification is a prerequisite for practice. Physicians undergo rigorous training, including undergraduate medical school, residency and often fellowship, prior to being granted a licence to practise. The professionalisation of medicine is supported by a wide range of knowledge, ethical codes and regulatory bodies such as the American Medical Association (AMA) in the USA and the General Medical Council (GMC) in the UK, which ensure that practitioners meet high standards of professional practice.

In contrast, healthcare management, which encompasses roles such as head of units or departments, executives of hospitals, and heads of departments in regional or national health authorities, does not always require a professional qualification. This is frequently observed in clinicians (healthcare professionals in direct contact with patients) transitioning into management. While there are academic programmes and certifications available in healthcare management and administration, many healthcare managers may come from a variety of professional backgrounds – most frequently, from a clinical background, while others may come from non-healthcare disciplines such as business and administration.^2^, ^3^, ^4^

The absence of a required professional qualification for healthcare management can result in large variations in the skills and knowledge possessed by those who have the responsibility to lead and manage healthcare organisations. The limited or absence of qualification and experience can impact the ability to effectively address the complexities of healthcare delivery and manage organisational change.^4^ Furthermore, it is important to differentiate between healthcare management and leadership. Management focuses on the administration and coordination of healthcare services, while leadership involves inspiring and guiding individuals and teams to achieve the organisation's goals and improve patient care. These skills work to complement each other.^2^, ^3^, ^4^

### How does healthcare management improve organisational results?

The research demonstrating the relationship between management capacities in the health sector and the organisational outcomes is recent and limited. Based on extensive literature review in 2003, the available evidence shows a link between managerial capacities and health services outcome.^5^ Nevertheless, because of a limited data base, small scale samples and most often empirical evidence, this conclusion is presented with caution.

The ‘management matters’ project launched in 2002 ambitioned to ‘measure the unmeasurable’. ‘The World Management Survey is the first cross-country dataset build to measure the quality of management practices in establishments’.^6^ Based on the framework developed for the management matters project, a survey^7^ on 1,200 hospitals in seven advanced countries demonstrated in 2011 that financial, clinical and patient-expressed outcomes are better in organisations with the best management scores. In addition, when comparing country by country the average scores in healthcare with other industries, they are always lower in the healthcare. More recently, in the context of emerging countries, the importance on investing more in management to achieve better performance was recognised.^8^

At the same time in the countries that are offering a full curriculum in health service management, an increased emphasis has been placed on the importance of developing competency-based education as an essential pillar for advancing the professionalisation of the healthcare management workforce.^9^ There are different models related to the competencies required for managing health organisations, but most of the models rely on similar core competencies that may be organised in different ways.^10^

In addition to the evidence on the benefit of proper management for achieving better outcomes, there are also other compelling arguments in the health sector to promote an increased role of management in all the layers of the health sector.

### The role of healthcare management on health service performance

The World Health Organization (WHO) Global Strategy on Human Resources for Health: Workforce 2030, published in 2016, identified a significant global shortage of healthcare workers, estimating an 18 million shortfall by 2030.^11^ The COVID crisis has exacerbated this worldwide shortage of health professionals,^12^ bringing to surface the major issue of the burnout of health professionals and linking it to excessive workload in organisations with shortcomings in human resource management.^13^ Addressing these challenges involves enhancing the capacity of healthcare professionals through skill acquisition, skill improvement and retention programmes, alongside developing ‘leadership pathways’.^14^ Considering the importance of human resources for delivering healthcare, it is paramount in such a global shortage context to have the capacity to optimise their contribution to health services. This requires that organisations that employ them are perfectly managed. For this, it is crucial to address the role of professional healthcare management.^14^ Added to this, there is a strong pressure for better utilisation of resources dedicated to healthcare^15^ in a context where countries are facing fiscal space constrains for the public spendings dedicated to healthcare. Improving the efficiency of healthcare spending requires better management of health services.^16^

### The competencies for the professionalisation of healthcare management

In several Organization for Economic Cooperation and Development (OECD) countries, the concern for enhancing management capacities has emerged more strongly in the beginning of the 21st century with a variety of initiatives. The UK medical sector takes great pride in creating the high standards that support clinical practice. Hence, the same level of thoroughness is applied to leadership and management in healthcare to maximise benefits for both patients and healthcare organisations. The leadership framework produced by the NHS Leadership Academy recognises five core leadership domains (demonstrating personal qualities, working with others, managing services, improving services, and setting direction) and two additional domains (creating the vision and delivering the strategy) that apply particularly, but not exclusively, to individuals in senior positional leadership roles.^17^ These leadership domains are applied at four levels of management and leadership competencies according to the position in an organisation. This approach is crucial for the full dissemination of leadership and management competencies. Recognising that at the team level there is already a need for mastering managerial and leadership capacities, furthermore, in 2011, the Faculty of Medical Leadership and Management (FMLM) was established.^18^

In the UK, leadership and management competencies are crucial for guiding the professional growth of medical leaders across career stages, emphasising the importance of adherence to evidence-based practices. In the health sector, all competencies that enhance emotional intelligence have an impact on health service delivery because all activities are related to interpersonal interactions within the teams caring for patients and their families. For example, ‘inclusion’ as a skill within the NHS leadership is identified as critical for enhancing patient care, organisational performance, and creating a psychologically safe workplace. Research has shown the negative impact of ‘exclusionary practices’ like bullying, which cost the NHS over £2 billion annually due to increased absenteeism, decreased productivity and compromised team effectiveness. This approach contrasts sharply with the traditional top-down bureaucratic behaviours, highlighting the need for systemic change towards more inclusive, compassionate leadership to address the deep-seated issues of fear, compliance and discrimination within the NHS.^19^

In Australia, as in all OECD countries, healthcare services operate in a highly complex health system. This system is characterised by influential professional groups, political influences and continuous change, particularly in medical technology, drug therapies and information systems. All these factors are also exacerbating the financial pressure on the health system and health leaders managing limited resources.^20^ This complexity has increased with a growing emphasis on patient safety, continuous quality improvement, and the need for a positive organisational culture. To navigate in such an environment, the Australasian College of Health Service Management (ACHSM) has established a leadership and management competencies model relying on five domains: leadership, health and healthcare environment, business skills, professional and social responsibility, and communication and relationship management.^21^ To better fulfil their responsibilities, clinician managers need to build capacities in each of the five domains. With such added capacities, clinician managers are expected to bring valuable contextual knowledge and credibility, which are essential for initiating change and understanding the professional subcultures within healthcare.^20^^,^^21^

In Canada, the LEADS framework also features five domains: lead self, engage others, achieve results, develop coalitions, and systems transformation.^22^ LEADS is a recognised leadership framework that encapsulates these qualities and supports the creation of healthy workplaces. Healthcare leadership is considered vital for guiding collaborative efforts towards health and wellness. It encompasses three main functions:^22^ First, it requires integrating services across various health systems and emphasising the need for cohesive and system-wide leadership. This includes physician leadership to overcome fragmentation. Second, effective leadership is crucial for fostering psychologically healthy and productive work environments. Good leadership is expected to promote optimism, trust and wellbeing among healthcare professionals, reducing burnout, absenteeism and workplace strain.^23^ Third, leadership drives necessary changes and reforms in healthcare, improving patient services and system efficiency. LEADS has become a benchmark for leadership in Canadian healthcare, aligning with medical education requirements and aiding in the professionalisation of healthcare leadership.^23^

In the USA, there are several recognised approaches in support of management professionalisation in healthcare. Interestingly, a consortium of six major professional membership organisations grouped in the healthcare leadership Alliance have collaboratively developed a competency model that is relevant and applicable to clinical, technical, nursing and administrative leadership.^24^ Based on these core competencies, the different professional associations have gone a step further with the credentialisation of individuals to recognise their specific competencies in health service management. The most advanced programme is the American College of Healthcare Executives (ACHE) that has formalised this approach in its fellowship programme.^25^ Such a programme is built on strict requirements demonstrating achievements in the field of leadership and management in addition to having a formal diploma in the field of management.

These examples from OECD countries are not exhaustive, as there are other similar initiatives that have been taken around the world in Latin American and Asian countries. They demonstrate that the concern for professionalisation of management in the health services is a growing trend and that the processes and tools for implementing it are available. In addition, there is also a growing concern to have the content of the healthcare management trainings to be recognised through a specific accreditation process.^26^

### The imperative of professionalisation in the eastern mediterranean region

In 2021, the WHO Regional Committee for the Eastern Mediterranean Region (EMR),quoted ‘Hospitals in the EMR are generally managed by medical doctors who often have no formal or informal management training and inadequate managerial skills’.^27^ The challenges in competency of management and leadership in healthcare in some of the EMR countries were highlighted further during the COVID-19 pandemic by the fragmentation of authorities and the multiplicity of actors involved, which lead to confusion and undermined public trust.^28^ Furthermore, learning from the pandemic, there's a need for effective human resource management which requires a blend of strategic planning, resource allocation, and the development of leadership competencies to ensure preparedness and resilience in healthcare systems​ in the EMR.^29^ The research on the importance of proper management practices to achieve organisational performance and the efforts made in various OECD countries illustrated earlier demonstrate that it is now possible to move away from the situation described in multiple parts of the EMR countries.

It is expected that, as the professionalisation of management evolves in the organisations, the expectations and the standards will follow a rising curve. At the same time, the dissemination of best practices will increase professionals’ confidence in making the most appropriate decisions and improve the trust from the public. This is clutching a continuous improvement dynamic that will benefit all. In this context, the concept of professionalisation of management takes its full meaning by having a career path organised among the health professionals for them to start with operational management responsibilities and grow in experience and competencies towards the responsibility to lead an organisation and ultimately to oversee a system.

### Balancing the ecosystem: approaches to leadership and management education

It is essential to recognise the shortcomings and the value of different kinds of management and leadership education. Sumantra Ghoshal critiques the instrumentalist focus of business schools in the global West, arguing that such approaches can destroy good management practices by neglecting the broader, more humanistic aspects of leadership.^30^ He suggests that the reductionist and overly analytical methods taught in many business schools fail to adopt the moral and ethical considerations necessary for effective management. Additionally, Ralph Stacey highlights that managerialist assumptions about predictability and control fail to account for the inherent complexity of organisations. He refers to the emergent nature of organisational behaviour, suggesting that traditional management theories are often insufficient for dealing with real-world complexities.^31^ Therefore, it is important to balance between education and practical learning.

This has been strongly demonstrated by Henry Mintzberg criticising the overemphasis given to the science of management versus the importance of peer-to-peer learning.^32^ Such an approach is fully aligned with the education of health professionals while giving a strong importance on practice-based learning.

Furthermore, Learmonth and Morrell contribute to this critique by illustrating that ‘leader’ and ‘follower’ are increasingly replacing ‘manager’ and ‘worker’ to become the routine way to frame hierarchy within organisations.^33^ A practice that blurs, even denies, structural antagonisms, such as many workers being indifferent to (and others despising) their bosses, by assuming that workers are ‘followers’ of organisational structure. This seems not only managerialist, but blind to other influential factors of cultural identity and constant sense making.^33^ This leads us to assume the importance of highlighting the practical aspects of leadership development further, shown by the 70:20:10 model created by McCall, Lombardo and Eichinger.^34^ This model posits that on-the-job experience and coaching are more beneficial for developing successful managers than formal education only. According to this model, 70 % of learning comes from job-related experiences, 20 % from interactions with others, and only 10 % from formal educational events. This highlights the importance of structured experiential learning and social interactions in developing effective leadership skills.^34^ Together, these perspectives share the need for a balanced approach to management and leadership education, integrating formal education with practical, ethical, and humanistic elements to prepare leaders for the complexities of organisational life.

### Recommendations

In the light of the review described and for the advancement of leadership and management recognised competencies, we would recommend for the EMR ten critical steps for moving towards effective professionalisation of healthcare management.

1. Establish standards: Set clear benchmarks and guidelines, including specific competency requirements, to define the roles and expectations of the individuals in the different levels and nature of responsibilities in the healthcare organisations. Such standards can be combining the international directory on core competencies^35^ with the Dreyfus model, which is about skill acquisition through distinct stages.^36^
2. Create a momentum: Advocating on the value and the importance of healthcare management to achieve better healthcare. This will generate a greater demand from the professionals in the field and make them realise their shortcomings, as well as that there is a gradation in the level of competencies required at the different levels of the organisation.
3. Provide education programmes: Develop rigorous undergraduate and graduate educational programmes in healthcare management to ensure that professionals have a strong foundation of knowledge and skills. Such a programme should preferably be accredited according to the most advanced standards for such education.
4. Offer an initiation to leadership and management in all the health professions’ training: Provide structured curriculum in their training programmes that cover key management aspects, ensuring that all acquire the minimum competencies to better understand what is at stake and how they should grow in competencies with their professional evolution and experiential experience.
5. Ensure certification systems: Implement a certification process to validate that healthcare professionals having managerial responsibilities meet the necessary competencies and standards acquired through both education and professional experiential development. Such certification can be granted by independent organisations or, as is the practice in the health sector, by respective professional associations.
6. Encourage continuous learning: A culture of lifelong learning through e-learning, workshops and educational programmes, ensuring that those with management responsibilities stay updated and maintain their competencies throughout their careers. This should be linked with the certification system to ensure periodic recertification requiring having earned a set number of educational credits over a period.
7. Encourage networks: Support the formation of professional associations dedicated to healthcare management and the dialogue between the associations to facilitate knowledge sharing and support, enabling to disseminate best practices and to learn from failures.
8. Influence policies: Advocate for policies that better recognise the importance of acquiring the proper management and leadership competencies for the various positions in health organisations. Support the development of undergraduate and graduate educational programmes in healthcare management, emphasising the importance of defined competencies regarding given responsibilities in health organisations.
9. Outline growth paths: Create transparent career pathways that align with the competency requirements acquired through education and training, to motivate professionals to advance in the field. Such advancement should break the silos of the initial professional education for managerial and executive position to be opened to all that have demonstrated their capacities and competencies in previous assignments.
10. Track progress: Implement a monitoring system to evaluate the effectiveness of these initiatives in developing and maintaining the required competencies. This system should integrate feedback from educational programmes and professional training. It should also favour a result-based approach with smart targets to be achieved at all levels and transparency on achievements.

Implementing these recommendations require resources and time, but first they need to be supported and acknowledged by the national political and professional leaders. These ten points offer a road map for action. Demonstrating that professionalisation of health service management is first about having a clear vision of what is needed to be accomplished and how it can be accomplished. When getting into the details of the implementation of these ten recommendations, a large part of the required resources can be leapfrogged from countries having advanced in the journey of health service management professionalisation. Last, it can be also argued that the resources that will be needed for these ten measures will contribute to enhance the performance of health services and therefore should be considered as an investment contributing to advancing the national health services for the population.

## Conclusion

In conclusion, the journey toward the professionalisation of healthcare management is not only essential but urgent. As the complexities of healthcare systems continue to grow, the gap between clinical expertise and managerial overview becomes increasingly evident. The examples from some of the OECD countries and the situation in the EMR stress a global consensus on the need for a structured and comprehensive approach to develop recognised competencies in management and leadership at all levels, as well as an increased professionalisation for those with the responsibility to lead the organisations. This includes globally setting standards, fostering education and training, ensuring certification, and promoting continuous learning. At the level of the executives of the organisations, healthcare management requires a full-time professional status, to ensure that healthcare services are led with the same level of expertise, ethics and commitment to excellence as the clinical activities. This professionalisation is not just for improving organisational efficiency and patient satisfaction, but also for enhancing the overall quality of healthcare delivery, demonstrating a global commitment to achieving the sustainable development goals on health and wellness.

## CRediT authorship contribution statement

**Qasem Al Salmi:** Conceptualization, Writing – original draft, Writing – review & editing. **Jehan Al Fannah:** Writing – original draft, Writing – review & editing. **Eric de Roodenbeke:** Writing – original draft, Writing – review & editing.

## Declaration of competing interest

The authors declare that they have no known competing financial interests or personal relationships that could have appeared to influence the work reported in this paper.
